# Low-dose cyclophosphamide combined with chinese herbal medicine Shuli Fenxiao formula for the treatment of intermediate-to-high risk primary membranous nephropathy

**DOI:** 10.3389/fimmu.2025.1543581

**Published:** 2025-04-22

**Authors:** Yiran Du, Yuning Liu, Zhiyan Chen, Yingnan Liang, Xia Li, Ying Wang, Jie Lv, Zhongjie Liu, Zhen Wang, Weihong Chen, Qingqing Liu, Xiaowen Li, Zhenjie Chen, Jingwei Zhou

**Affiliations:** ^1^ Department of Nephrology, Dongzhimen Hospital, Beijing University of Chinese Medicine, Beijing, China; ^2^ Xiyuan Hospital, Department of Nephrology, China Academy of Chinese Medical Sciences, Beijing, China

**Keywords:** membranous nephropathy, therapeutic, side effects, integrative medicine, Shuli Fenxiao formula

## Abstract

**Background:**

At present, the side effects of hormonal and immunosuppressant therapy for intermediate-to-high risk primary membranous nephropathy (PMN) are relatively large, and the remission rate is limited, so more safe and effective regimens are needed.

**Methods:**

This study is a clinical prospective case series study. 31 patients were finally included. The intervention was cyclophosphamide (CTX) combined with “Shuli Fenxiao formula(SLFX formula)”, and the patients were treated for 24 weeks. The observation nodes were baseline, 2 weeks、12weeks and 24weeks after treatment.

**Results:**

At 12 weeks of treatment, 38.7% of patients achieved partial response. At 24 weeks of treatment, 61.3% of patients achieved partial response and 24.5% achieved complete response. All Anti-phosholipase A2 Receptor Antibody (Anti-PLA2R) seropositive patients achieved immune remission. 24-hour urine total protein quantification (24hUTP) decreased from a median of 6.1 (IQR, 4.6-8.4) g/d to 2.7 (IQR, 0.6-8.7) g/d (P<0.001). Serum albumin (ALB) increased from 27.2 ± 6.4 g/L to 31.9 ± 8.0 (P<0.05). Within 24 weeks of follow-up after discharge, no patients relapsed. During the treatment follow-up period, 6 adverse events occurred in 31 patients, 1 patient developed heart failure during the treatment period, which was not considered to be clearly associated with treatment regimen or nephrotic syndrome (NS), 3 patients were infected, and 2 patients had liver impairment.

**Conclusion:**

The results suggest that the combination of CTX and SLFX formula dramatically decreased Anti-PLA2R titers and 24hUTP levels, increased ALB in short term. The combination was safe and had few adverse effects. It has the potential to be used as a potential option for the clinical treatment of intermediate-to-high risk PMN patients, particularly for elderly patients with contraindications to corticosteroid use or those with refractory disease.

**International Traditional Medicine Clinical Trial Registry:**

http://itmctr.ccebtcm.org.cn/, identifier ITMCTR2025000355.

## 1 Introduction

Primary membranous nephropathy (PMN) is a renal-confined autoimmune disease which is the leading cause of nephrotic syndrome (NS) in adults, accounting for approximately 30 percent of cases (1). According to epidemiological statistics, the prevalence of PMN has risen by 13% in recent years, and this increase is linked to environmental pollution and declining population size (2). Proteinuria is the primary clinical symptom of PMN, and the primary pathological alterations include the formation of immune complexes between target antigens and autoantibodies on the glomerular podocyte membrane, which further thickens the basement membrane, damages the podocytes, and ultimately destroys the glomerular filtration barrier. This pathologic damage is irreversible. As the disease progresses, approximately two-thirds of patients with PMN will develop end-stage renal disease after 20 years, severely affecting the life expectancy of patients (3). Anti-PLA2R antibody titer is a critical biomarker for assessing disease activity in membranous nephropathy. With effective treatment, the antibody titer progressively declines, and immunological remission generally occurs earlier than clinical remission (4). Following the achievement of immunological remission, rigorous monitoring of proteinuria and renal function is essential to evaluate the likelihood of the patient attaining clinical remission (5).

Currently, the optimal treatment plan for patients with PMN is determined by the various risk categories. The Global Organization for Improving the Prognosis of Kidney Disease (KDIGO) (6) proposes in the clinical practice guidelines for kidney disease that patients with PMN should be risk-stratified based on renal function, proteinuria quantification and antibody titers. About one-third of individuals with low-risk PMN who receive regular testing and basic supportive care will have spontaneous remission. Hormonal or immunosuppressive medication is necessary to manage the progression of the disease in high-risk PMN patients and intermediate-risk PMN patients with a progressive increase in proteinuria. The glomerular filtration barrier will be severely damaged as a result of the ongoing high levels of proteinuria and titer antibody levels, increasing the risk of renal failure.

For intermediate-to-high risk PMN, the first-line treatment is long-term cyclophosphamide (CTX) combined with glucocorticoid (GC). The combination of CTX and GC reduces the risk of end-stage renal disease by inhibiting immunity, alleviating renal immune damage, reversing the development of renal sclerosis and fibrosis, and inducing long-term remission of kidney disease (7). Even in patients receiving low-dose CTX 1.5 mg/kg/day, CTX with GC has been clinically shown to be quite hazardous (8). The prognosis and treatment compliance of PMN patients are significantly impacted by the potential for severe adverse responses, such as liver damage, bone marrow suppression, gonadal suppression, and malignancies, that can result from long-term use of CTX in conjunction with GC.

Due to the severe adverse effects of combination between CTX and GC, current studies have proposed steroid-free hormone regimens for patients with high-risk PMN. Xing HL. et al. (9) found that the low-dose rituximab (RTX) combining with low-dose tacrolimus (TAC) regimen induced serologic remission earlier than the classical regimen of GC combining with TAC in patients with intermediate-to-high risk PMN, with no significant difference in adverse effects between the two groups. Studies have shown that PMN is ineffective with both GC and CTX alone, and the combination of CTX is 50%-60% effective (10). Compared to the combination, CTX alone lowers the risk of opportunistic bacterial, fungal, and viral infections; however, long-term use of the medication increases the risk of thrombocytopenia, gonadal suppression, and unpleasant malignancies like leukemia, hemorrhagic cystitis, and bladder cancer (11).

In our previous study, we found that the clinical use of Shuli Fenxiao formula (SLFX formula) in the treatment of intermediate-risk PMN patients could reduce proteinuria and increase serum albumin (12). In animal experiments, it was found that “SLFX formula” is likely to reduce LC3, P62, and unblock the autophagy pathway, thereby counteracting the damage of podocytes and slowing down the progression of the disease (13). Based on this, this study used “SLFX formula” to replace GC to conduct a prospective study in patients with intermediate-to-high risk PMN to explore the efficacy and side effects of “SLFX formula” combined with low-dose CTX.

## 2 Materials and methods

### 2.1 Study design

This study was a real-world prospective own before-and-after controlled study that selected PMN patients who attended outpatient clinics or wards of Dongzhimen Hospital, Beijing University of Traditional Chinese Medicine, between May 2021 and December 2023, and screened and enrolled all intermediate-to-high risk PMN patients who met the criteria. The study was approved by the Ethics Committee of Dongzhimen Hospital with the ethical approval number 2021DZMEC-030-01, and all included patients gave informed consent and signed the informed consent form.

### 2.2 Inclusion and exclusion criteria

Inclusion Criteria: All patients were diagnosed with biopsy-proven PMN or serum Anti-PLA2R positivity. Patients met the following criteria prior to initiating combination therapy: 1) those aged 18-80 years; 2) estimated glomerular filtration rate (eGFR) > 30 ml/min/1.73 m^2^; 3) a pathologically confirmed diagnosis of membranous nephropathy (stage 1-4) or Anti-PLA2R positivity by renal puncture biopsy; 4) met the diagnostic criteria for intermediate-to-high risk PMN; reference was made to the KDIGO Practice Guidelines for Clinical Management of Glomerular Diseases, 2021, and the Up to date guidelines, evaluated on the basis of temporal trends in clinical and serologic parameters; 5) patients who had received Renin Angiotensin System Inhibitor (RASi) for more than 3 months and still had 24-hour urine total protein quantification (24hUTP) quantification > 3.5 g/d; 6) no prior treatment with hormones and other immunosuppressive agents.

Exclusion Criteria: 1) pregnant, breastfeeding, or had a plan to become pregnant in the near future; 2) needed corticosteroid immunotherapy; 3) other types of MN, such as rapidly progressive membranous nephropathy (50% decrease in eGFR within 3 months) and secondary membranous nephropathy (SMN), such as infection, autoimmune disease, drugs, tumors, heavy metals, and so on; 4) who had used or were using steroids or other immunosuppressants in the last 6 months; 5) with heart failure, abnormal liver function, abnormal coagulation function, and current infection; 6) who were allergic to the study drugs; 7) with serious mental illness or with the presence of steroids or other immunosuppressants; 8) malignant/uncontrollable high blood pressure; 9) other reasons for the current unstable renal function, such as acute renal injury caused by massive hematuria; 10) white blood cell count < 3.0×10^9^/L or other blood disorders; 11) electrocardiogram showing prolonged QT interval or severe arrhythmia, or concomitant use of medications that may cause prolongation of the QT interval.

### 2.3 Treatment

In order to suppress the renal immune response, all patients received oral CTX 50 mg once daily for 24 weeks. The Chinese herbal medicine was administered in the form of SLFX formula, 250 ml twice daily with hot water, from the outpatient pharmacy of Dongzhimen Hospital of Beijing University of Traditional Chinese Medicine (BUTCM). The **Supplementary Material** details the precise composition of the Chinese herbal medicines, the dosage, and how it was adjusted based on the patient’s condition.

During the medication period, blood pressure and blood lipids of the patients were kept as under control as feasible, and appropriate basic treatment was given according to the KDIGO clinical practice guidelines. This included: 1) adequate dietary protein (0.8-1.0 g/kg/day), adequate caloric intake, and a low-salt diet (<3 g/day); 2) administration of RASi to control blood pressure around 125-130/75-80 mmHg; 3) use of statins (HMG CoA reductase inhibitors) to control the patient’s hyperlipidemia; 4) if serum albumin (ALB) is below 20-25g/L or there are other risk factors for thrombosis, then anticoagulation with low molecular heparin or warfarin is used.

### 2.4 Outcome measures

#### 2.4.1 Primary outcome

The primary outcome was a composite of complete remission and partial remission. According to the 2021 KDIGO guidelines: 1) complete remission is defined as: 24hUTP < 0.3g/d, serum ALB > 35g/L, and normal renal function: 2) partial remission is defined as: 0.3g/d < 24hUTP < 3.5g/d or > 50% decrease from baseline levels, significant improvement in serum ALB, and stable renal function; and 3) failure to remit is defined as: failure to achieve the above criteria, with 24hUTP > 3.5g/d, < 50% decrease from baseline level, and worsening renal function.

#### 2.4.2 Secondary outcome

Secondary outcomes included changes in 24hUTP, serum creatinine (Scr), ALB, cholesterol (CHOL), low density lipoprotein (LDL-C), and triglyceride (TG) levels from baseline to 24 weeks after the start of treatment. Changes in serum Anti-PLA2R titers and relapse rates were also assessed. Immune remission was defined as ELISA-negative serum Anti-PLA2R titers decreased by more than 50% compared to the baseline value. Relapse was defined as a 24hUTP > 3.5 g/d after achieving complete or partial remission. All relapses were reviewed to differentiate clinically insignificant fluctuations in 24hUTP values from clinical relapses.

#### 2.4.3 Serious adverse events

Serious adverse events including events such as hospitalization or life-threatening or organ damage. Follow-up was performed during the 24-week treatment period. Patient’s blood and biochemical test results were regularly reviewed to assess for events such as serious infections, hematological disorders, liver and kidney impairment.

### 2.5 Monitoring nodes

All patients were monitored from the start of combination therapy (initial administration of CTX and SLFX formula) until the date of the outpatient visit at the last dose of therapy. The monitoring points were at the baseline visit, 2 weeks later, 12 weeks later, and 24 weeks later, with routine blood and liver function tests at 2 weeks to assess safety, and the following laboratory values at the 12-week and 24-week visits: routine blood, full biochemistry, and urine protein quantification.

### 2.6 Statistical analysis

The Case Report Form(CRF) table was designated for data collection and statistically was performed using STATA 18.0 software, with *P* < 0.05 considered statistically significant. Descriptive statistics were performed for the general information and baseline clinical indicators of the included patients, with continuous variables expressed as median and interquartile range (IQR) or mean and standard deviation, as appropriate, and categorical variables expressed as frequency and percentage. For continuous information, data satisfying normal distribution were compared within groups using t-tests. As appropriate, data that did not satisfy normal distribution were compared between groups using the nonparametric Wilcoxon test. Categorical data were compared between components using the chi-square test. If the data met the overall distribution, they were analyzed using the bivariate Pearson linear correlation test. If data did not conform to normal distribution, Spearman correlation analysis was used. Baseline parameters were compared using the Wilcoxon rank sum test or the Fisher exact test. Kaplan-Meier method was used to examine time to partial remission and time to complete remission from first enrolment. If the primary outcome was not achieved during the study period, patients were reviewed at the last follow-up date. Differences between Kaplan-Meier curves were assessed using the log-rank test and the Cox proportional risk model, and all comparisons were two-tailed.

## 3 Results

### 3.1 Baseline data information

From May 2021 to December 2023, a total of 31 patients with intermediate-to-high risk PMN in our wards and outpatient clinics were enrolled in this study. Patients with membranous nephropathy had been definitively diagnosed by renal puncture pathological biopsy or underwent a positive serum Anti-PLA2R test. The baseline Scr distribution was 67.5 (IQR, 57.5-84.1) umol/L, eGFR was 101.3 (IQR, 89.4-115.3) ml/min·1.73m^2^, 24hUTP distribution was 6.1 (IQR, 4.6-8.4) g/d, serum ALB distribution was 27.2 ± 6.4 g/L, and Anti-PLA2R antibody titer distribution was 73.2 (IQR, 9.3-298.5) RU/ml. Specific baseline data information is shown in **Table 1**. Combined with serological data and renal puncture biopsy, 80.6% of patients were diagnosed with PLA2R-associated PMN. 19 patients underwent renal biopsy for definitive diagnosis, with the largest proportion of patients with stage I PMN, 22.6%, and the specific information is shown in **Table 1**.

**Table 1:** Baseline clinical characteristics of participants.

Variable	Total
No. of patients	31
Age, y	50.6 ± 14.5
Female sex	10 (32.3%)
Systolic BP, mm Hg	139.7 ± 18.8
Diastolic BP, mm Hg	82.9 ± 9.0
Serum creatinine, μmol/L	67.5 [57.5,84.1]
eGFR(mL/min/1.73m^2^)	101.3 [89.4,115.3]
24hUTP(g/d)	6.1 [4.6,8.4]
24hUTPcategory	
<4g/d	6 (19.4%)
4-8g/d	16 (51.6%)
>8g/d	9 (29.0%)
Nephrotic syndrome	27 (87.1%)
Albumin, g/L	27.2 ± 6.4
Uric acid, μmol/L	389.5 ± 88.0
Triglycerides, mmol/L	2.1 [1.6,4.3]
Low density lipoprotein, mmol/L	4.8 [3.1,6.6]
Total cholesterol, mmol/L	7.2 [5.8,8.6]
PLA2R-associated disease	
Yes(n, %)	25 (80.6%)
No(n, %)	4 (12.9%)
Unknown(n, %)	2 (6.5%)
Diagnosis of renal puncture biopsy	
Yes (n, %)	19 (61.3%)
No (n, %)	12 (38.7%)
Phase I	7 (22.6%)
Phase I-II	2 (6.5%)
Phase II	4(12.9%)
Phase II-III	1 (3.2%)
Atypical	1 (3.2%)
Anti-PLA2R antibody titer, RU/mL	73.2 [9.3-298.5]

Normally distributed data are represented as mean ± standard deviations, non-normally distributed data are presented as median [interquartile range], and frequency data are expressed as n (%). Nephrotic syndrome is defined as 24hUTP > 3.5 g/d, ALB < 30 g/L, and peripheral edema.

### 3.2 Primary outcome

#### 3.2.1 Effect of the combination regimen on response rates in the short term

**Figure 1:**
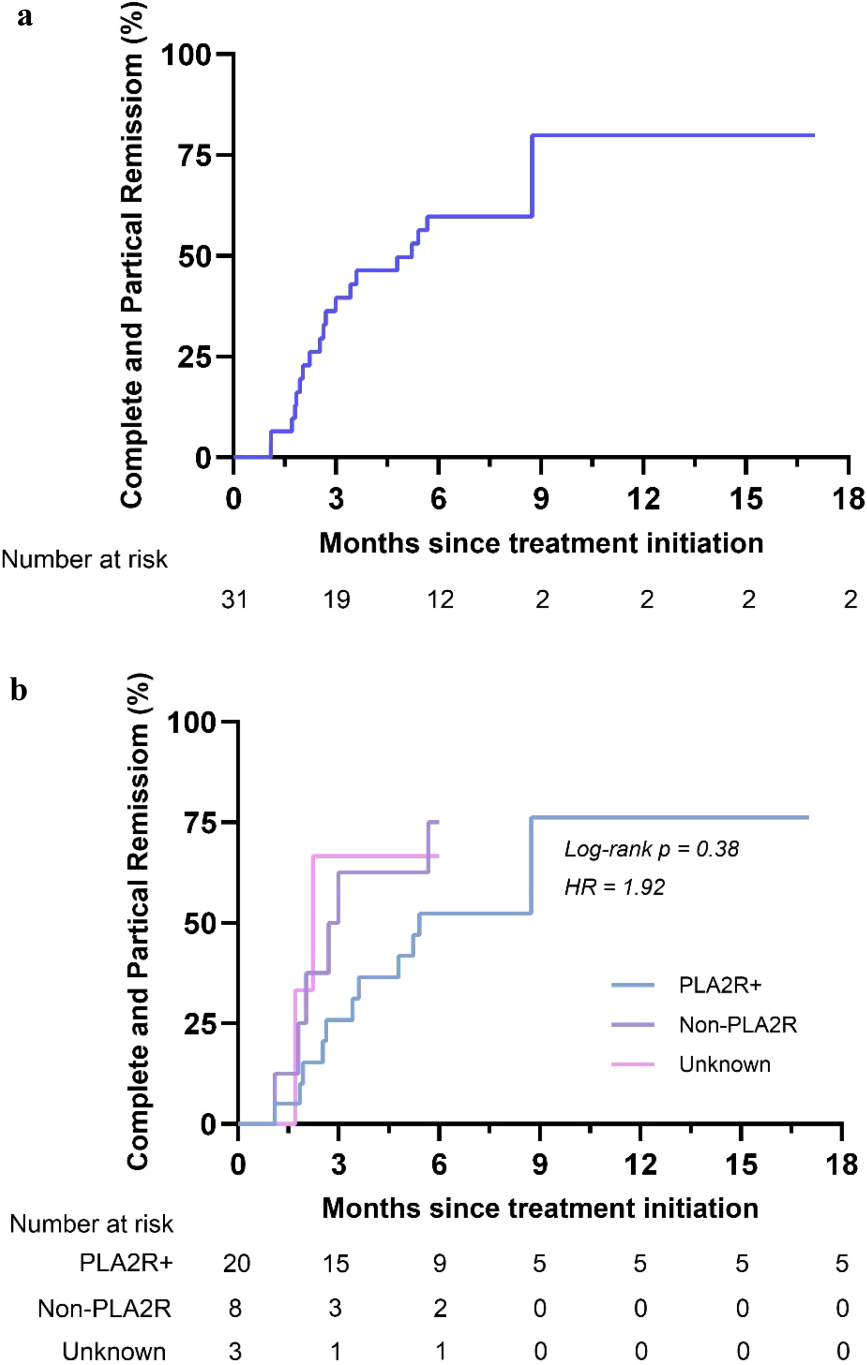
Kaplan-Meier curves for partial and complete remission. Kaplan-Meier curves for the overall group **(a)** and stratified by 24hUTP **(b)**.

As shown in **Table 2**, of the 31 patients who received CTX in combination with SLFX formula, the efficiency was 38.7% at 12 weeks and 64.5% at 24 weeks. During the study period, 19 patients achieved complete remission or partial remission. The median time to achieve remission was 4.8 (IQR, 2.0-6.0) months (**Figure 1a**). 8 patients achieved complete remission during the study period, and all of the patients who achieved complete remission had a 24hUTP < 0.3g/d and serum ALB > 35g/L during the treatment period.

**Table 2:** Response rate of participants at 3, 6 months.

Time	CR	PR	NR	Efficiency
12 w	1 (3.2%)	11 (35.5%)	19 (61.3%)	12 (38.7%)
24 w	8 (25.8%)	11 (35.5%)	12 (38.7%)	19 (61.3%)

CR, complete response after treatment; PR, partial response after treatment; NR, no response after treatment.

Stratification of patients according to 24hUTP, Anti-PLA2R and renal pathology levels revealed differences in time to remission across strata. Among them, patients with lower baseline proteinuria levels had shorter time to remission (hazard ratio = 4.85, log rank *P* = 0.03 < 0.05) (**Figure 1b**). There was no difference in time to complete remission when stratified by anti-PLA2R status (hazard ratio = 1.92, log rank *P* = 0.38) (**Supplementary Figure S1**). Time to remission was shorter in patients with less severe renal pathological stage (hazard ratio = 6.75, log rank *P* = 0.03 < 0.05) (**Supplementary Figure S2**).

**Table 3** summarized the changes in Scr, serum ALB, 24hUTP, and Anti-PLA2R antibodies in the 11 patients who failed to achieve remission during the study period, and although proteinuria remission was not achieved, all serum Anti-PLA2R-positive patients achieved immuno-serological remission after 24 weeks of treatment.

**Table 3:** Patients who did not achieve clinical remission.

Patient	Sex	Age	PLA2R Association	Cr (μmol/L)		ALB (g/L)		24hUTP (g/d)		PLA2R (RU/ml)		Immunologic remission^b^	Clinical outcome
				Initial^a^	End^a^	Initial	End	Initial	End	Initial	End		
1	M	60	Y	80.5	66.9	21.2	20	6.0	8.7	419.2	253	Y	NR
2	F	78	Y	143.9	178	20.8	21.4	10.7	15.8	397.6	6.87	Y	NR
3	M	25	Y	72.9	64.6	16.2	16.9	8.8	10.1	165.9	87.2	Y	NR
4	M	37	Y	84.1	56.7	23.2	22.2	6.7	6.7	83	43	Y	NR
5	M	35	N	59.0	58.9	33.2	33.7	8.3	8.8	n/a	n/a	n/a	NR
6	M	37	N	69.0	72.0	35.4	34	3.9	3.6	131	–	–	NR
7	F	51	Y	64.6	59.5	19.8	20.4	16.2	12.0	557.7	34.4	Y	NR
8	M	74	Y	65.7	91	28.2	30.9	5.7	5.4	206.9	23.4	Y	NR
9	M	69	N	133	129	26.5	31.5	5.4	5.9	n/a	n/a	n/a	NR
10	M	64	Y	85.5	68.8	25.2	25.7	12.3	12.9	127.7	40.7	Y	NR
11	M	59	Y	78.6	88.4	23.9	23.5	12.2	8.8	63.4	–	n/a	NR

*M*, male; *F*, female; *Y*, yes; *N*, no; *Cr*, creatinine; *ALB*, Serum albumin; *n/a*, not applicable; *24hUTP*, 24-hour urine total protein quantification; *PLA2R*, phospholipase A2 receptor; *NR*, non-remission. a. Initial values represent values at the start of treatment; End values represent values at last follow-up. b. Immunologic remission is defined as serum Anti-PLA2R titers decreased by more than 50% compared to the baseline value.

### 3.3 Secondary outcomes

#### 3.3.1 Effect of the combination regimen on nephrotic syndrome parameters

**Figure 2:**
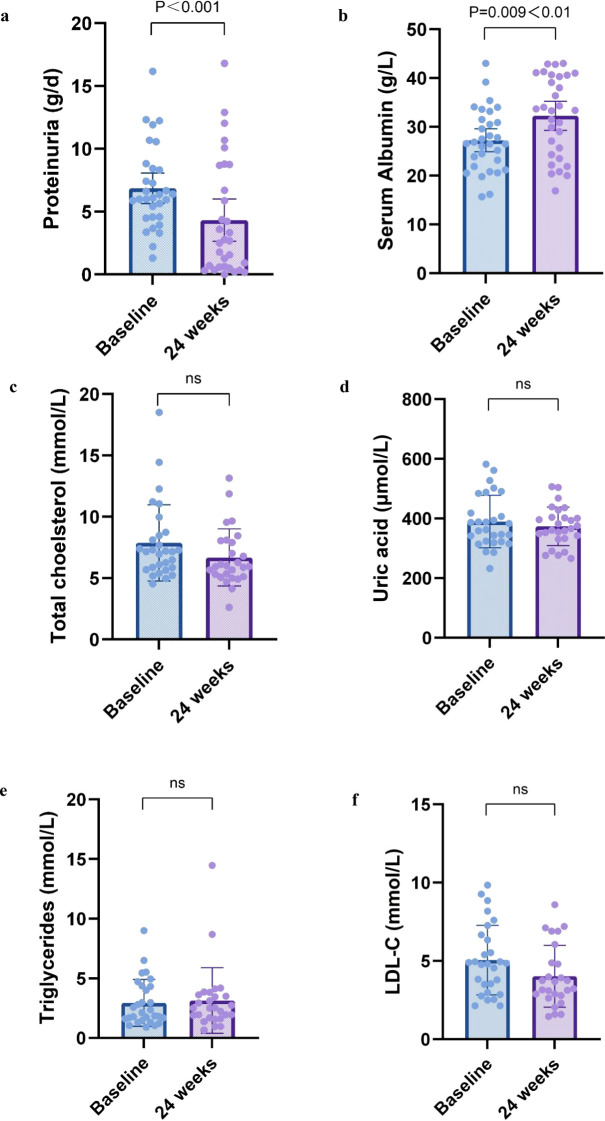
Nephrotic syndrome parameters at 6 months. Change in **(a)** proteinuria, **(b)** serum albumin, **(c)** total cholesterol, **(d)** uric acid, **(e)** triglycerides, and **(f)** ldl-c from baseline to 12 months. All box and whisker plots represent median (IQR) and the minimum-maximum range measurements. Longitudinal differences from baseline to 12 months were analyzed with the Wilcoxon signed-rank test.

Changes in nephrotic syndrome parameters in patients after 24 weeks of treatment were shown in **Figures 2a–f.** For nephrotic syndrome, 24-hour urine protein quantification decreased from a median of 6.1 (IQR, 4.6-8.4) g/d to 2.7 (IQR, 0.6-8.7) g/d (*P<0*.001). Scr increased from 27.2 ± 6.4 g/L to 31.9 ± 8.0 (*P=0*.009<0.05). There was a downward trend in total CHOL and LDL from baseline, and the difference was not statistically significant (P>0.05).

#### 3.3.2 Effect of the combination regimen on renal function parameters

**Figure 3:**
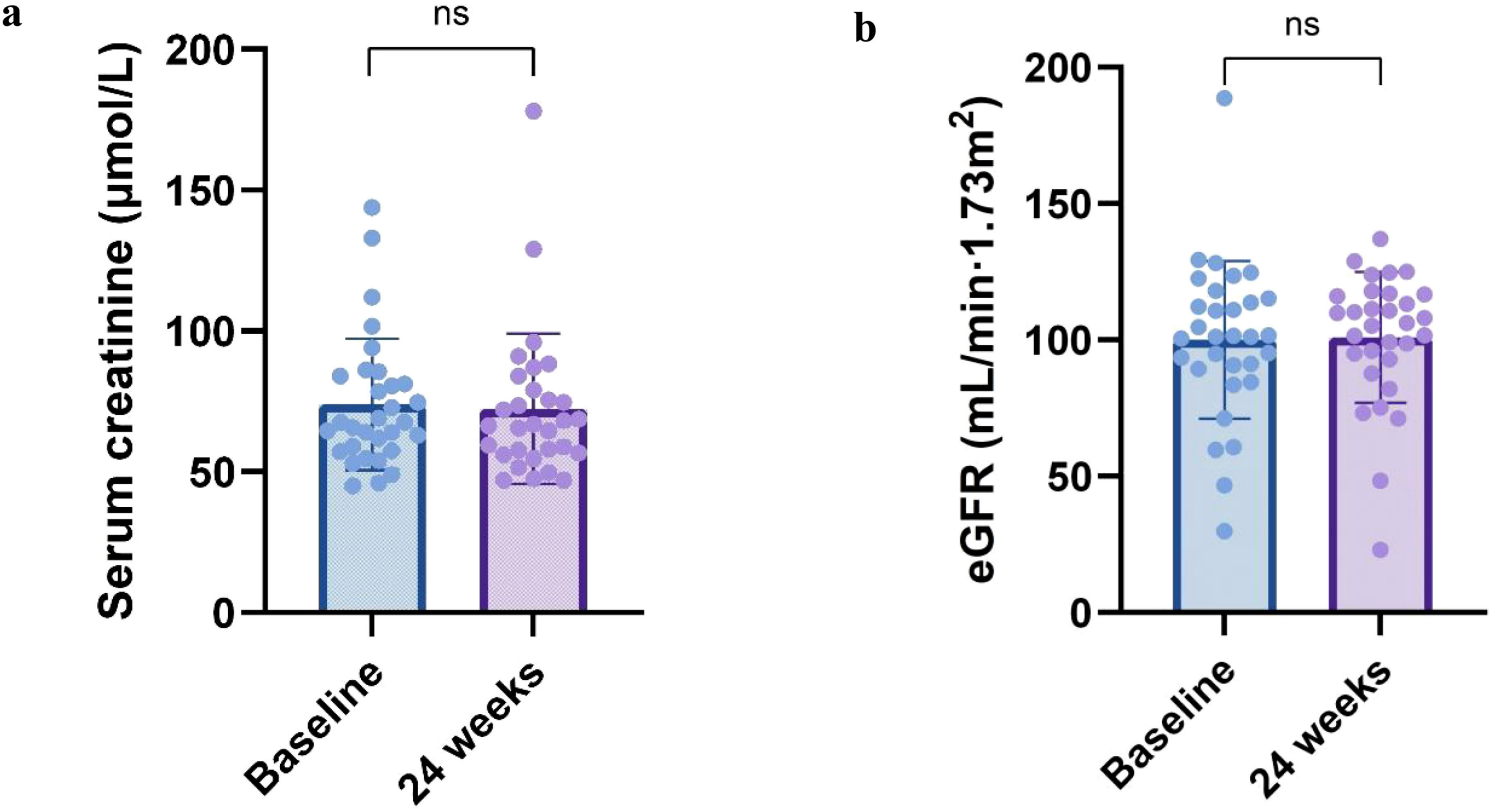
Renal function parameters at 24 weeks. Change in **(a)** serum creatinine, **(b)** eGFR from baseline to 24 weeks. All box and whisker plots represent median (IQR) and the minimum-maximum range measurements. Longitudinal differences from baseline to 12 months were analyzed with the Wilcoxon signed-rank test.

Changes in renal function of patients before and after treatment were shown in **Figures 3a, b**. Although the Scr showed a decreasing trend and the estimated glomerular filtration rate both increased after treatment compared to before, the difference between before and after treatment was not statistically significant in both cases (*P* > 0.05).

#### 3.3.3 Effect of combined regimen on serum PLA2R antibody titers

**Figure 4:**
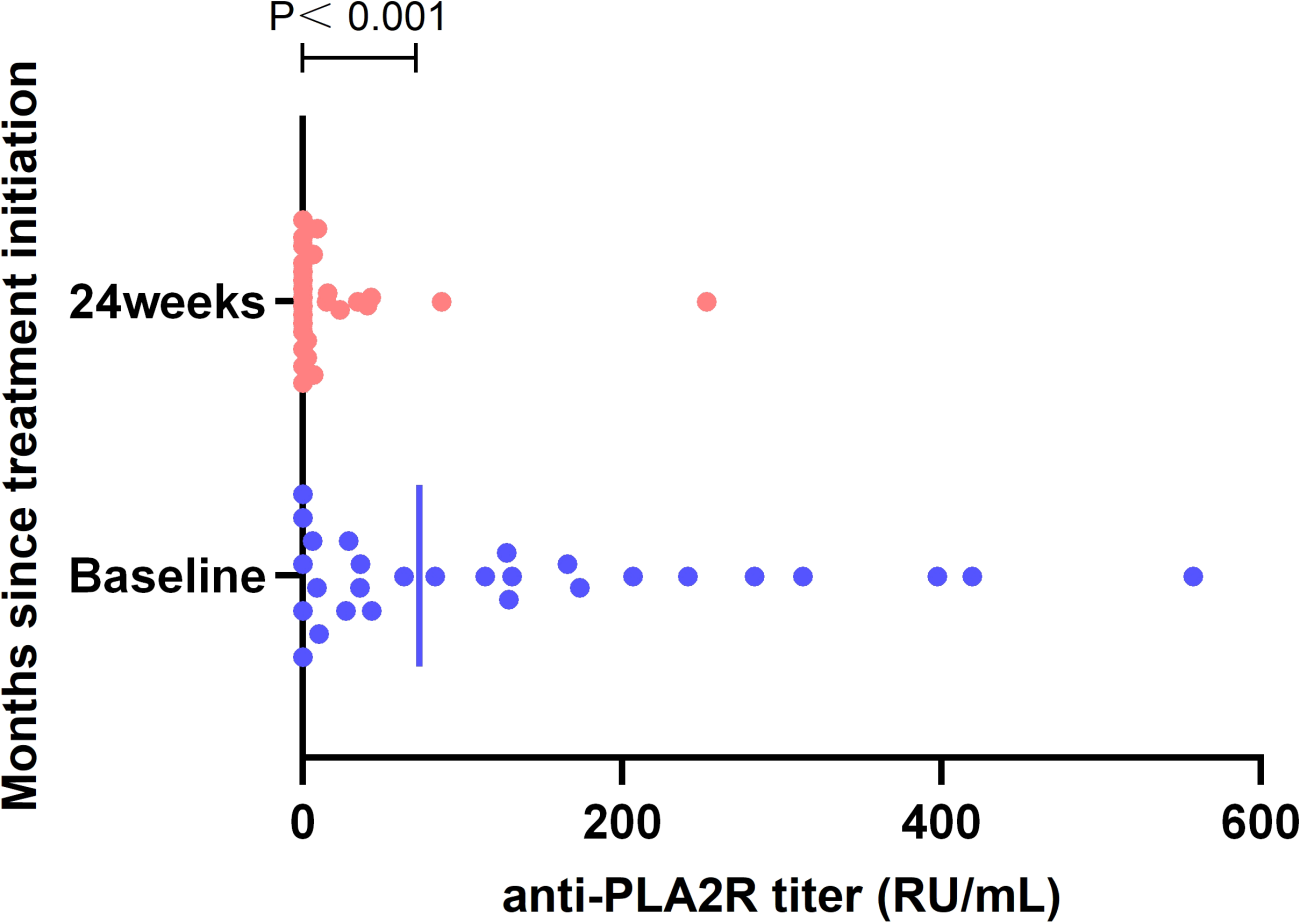
Anti-PLA2R titers with treatment. There is a significant decline in titer of anti-PLA2R antibodies over time compared to baseline. ELISA reference ranges: negative, <14 RU/mL; borderline, 14-20 RU/mL; positive, > 20.0 RU/mL. ELISA, enzyme-linked immunosorbent assay; IQR, interquartile range; PLA2R, phospholipase A2 receptor; RU, relative units.

Serum Anti-PLA2R antibody titers were shown in **Figure 4**, and after 24 weeks of treatment, all patients (*n=*20) who were seropositive for Anti-PLA2R at baseline achieved immune remission. Anti-PLA2R antibody titers before and after treatment were 73.2 (IQR, 9.3-298.5) and 0.0 (IQR, 0.0-15.5) IU/ml, respectively, which were significantly different (*P<0*.001).

### 3.4 Adverse events

Serious adverse events were listed in **Table 4**. Five serious adverse events were identified during the combined follow-up time of 31 patients. The most common adverse events were CTX-induced infections (*n=*3), and hepatic impairment (*n=*2). None of the patients experienced deterioration of renal function and the need for renal replacement therapy during the treatment period. Also no patient developed malignancy.

**Table 4:** Serious adverse events.

Adverse Event	No. of Events
Lung infections	1
Heart failure	1
Urinary tract infections	2
Abnormal liver function	1
Nausea, diarrhea	1

## 4 Discussion

Pathological damage of PMN is mainly manifested as the destruction of the glomerular basement membrane. In the pathological progression of PMN, immune complexes are deposited in the glomerular podocyte membrane, resulting in the destruction of the glomerular basement membrane. At this point, the patient has a small amount of proteinuria leaking, which is at low risk of PMN. With the increase of basement membrane destruction, protein leakage increases, and more serious pathological damage such as sclerosis and fibrosis occur in the kidneys, the patient is in the intermediate-to-high risk stage, with high Anti-PLA2R antibody levels and massive proteinuria. Patients in the intermediate-to-high risk groups usually have severe and irreversible pathological injuries, which have a greater impact on the development of glomerular fibrosis and are at high risk of progression to end stage kidney disease(ESKD) (14). The KDIGO guidelines recommend long-term treatment with immunosuppressants in combination with GC to prevent the irreversible damage to the glomerular basement membrane from worsening on the basis of conventional treatment.

The initial therapy for high-risk PMN has been CTX with GC. Clinical studies have shown that immunosuppressive regimens for membranous nephropathy typically have a duration of response of more than six months (15). However, during long-term treatment with CTX combined with GC, patients often have adverse reactions such as infection, liver insufficiency, cytopenias, and gastrointestinal disorders, which makes it difficult for patients to adhere to treatment. A study primarily assessing safety outcomes found that patients treated with CTX and GC had a higher incidence of adverse events in PMN compared with RTX monotherapy (16), with infection, leukopenia, and Cushing syndrome being more common. Severe infection, as a common adverse reaction of GC combined with CTX, is the main cause of disease progression and death in patients with MN. Patients with MN often have hypoproteinemia and low serum IgG levels. In addition, immunosuppressants and hormone therapy can impair the patient’s innate immune, cellular immunity, and humoral immunity. Together, these factors increase the risk of infection, including conditionally pathogenic bacteria, fungi, and viruses (17).

Although steroid-free therapy has been proposed to improve renal impairment in patients, CTX alone still has side effects such as liver function impairment and hematologic impairment (18–20). Patients with PMN treated with CTX have a three-fold higher incidence of malignancy compared with those who do not receive CTX (21). For example, in the STARMEN study (22), 4 of 5 serious infections (80%) occurred in the CTX-GC group, and in the RI-CYCLO study (23), 5 patients (14%) in the CTX-GC group had serious adverse events, mainly leukopenia and pneumonia. These side effects are associated with myelosuppressive and immunosuppressive mechanisms of CTX.

In previous studies, we found that SLFX formula can attenuate the toxicity of immunosuppressants by reducing inflammatory expression, inhibiting apoptosis, regulating immune response, and other multi-target and multi-pathways (24). Simultaneously, during clinical practice, it was shown that patients with PMN tended to have much lower urine protein, higher serum albumin, and less edema (12).

Radix Astragali (Astragalus membranaceus polysaccharides), Bupleurum (saikosaponin), Rhizoma smilacis glabraea cocos, Scutellaria, and other active ingredients of traditional Chinese medicine have been found to be the main drugs of SLFX formula in pharmacological studies (25–27). These ingredients have detoxifying and synergistic effects on CTX, including anti-inflammatory, antioxidant, anti-fibrotic, immune system regulation, and other effects.

In this study, a prospective trial was conducted to explore the short-term clinical efficacy of SLFX formula combined with CTX in patients with intermediate-to-high risk PMN. In this study, we used the lowest dose of CTX (50 mg qd) for the same patient’s treatment regimen with a cumulative CTX dose of 9 g, and we found that after 24 weeks of CTX combined with traditional Chinese medicine, the clinical remission rate of patients with PMN reached 64.5%, and the treatment time required was shorter for patients with lower urine protein levels and milder pathologic stages. The parameters of NS in all patients improved after treatment, which was manifested by a decrease in 24-hour urine protein quantification and an increase in serum ALB expression. However, there was no significant change in renal function (as measured by serum Scr and eGFR). In addition, serum Anti-PLA2R antibodies decreased during treatment, with a total of 13 patients (65%) turning negative with Anti-PLA2R antibodies after 6 months of treatment.

In order to better analyze the therapeutic effect of SLFX formula combined with CTX, we selected large RCTs in the treatment of membranous nephropathy, including the STARMEN trial (22) and the RI-CYCLO trial (23), and compared the clinical outcome criteria of CTX plus GC in the treatment of CTX in terms of clinical response, immune remission, and relapse, as shown in **Table 5**. We found that the short-term response rate in this study was slightly inferior to that of CTX-GC in the absence of hormones. Considered to be related to: 1) The short duration of treatment in this study and the lack of follow-up. Previous studies (28) have shown that the duration of treatment and follow-up of MN disease is 12-24 months, and the response rate may further increase with the extension of treatment and follow-up time. 2) Serum Anti-PLA2R antibody levels (29) have been shown to be an early predictor of late proteinuria remission, as well as the risk of renal outcome remission and progression to ESKD. The patients included in this study were patients with intermediate-to-high risk membranous nephropathy, with a large basal 24hUTP quantification and Anti-PLA2R antibody titers, a longer course of disease remission, and a slightly higher remission rate than expected. 3) According to previous studies (30, 31), it is difficult to achieve clinical efficacy in the treatment of single-agent CTX or GC in the treatment of membranous nephropathy, and the addition of traditional Chinese medicine on the basis of single-agent CTX in this study has improved the clinical effective rate and reduced the side effects of hormones, so it is considered that this regimen has certain efficacy for patients with intermediate-to-high membranous nephropathy. In addition, the combination of traditional Chinese medicine with CTX has improved the side effects of CTX monotherapy under the condition of certain clinical efficacy, suggesting that this regimen can be used in the treatment of PMN patients for a long time in clinical practice.

**Table 5:** Comparison of basic information and mitigation information between three studies.

Variable		This Study	STARMIN	RI-CYCLO
Regimen		SLFX formula-Cyclophosphamide	Corticosteroid-Cyclophosphamide	Corticosteroid-Cyclophosphamide
Duration		24 w	12 m	12 m
Initial PLA2R(+)		25 (31)	29 (37)	46 (71)
Initial 24hUTP		6.1 [4.6,8.4]	7.4 [4.8–11.3]	1.7 ± 0.5
Clinical response(%)	12w	38.7	51.2	/
	24w	61.3	74.4	65
Complete response(%)	12w	3.2	2.3	/
	24w	25.8	12.2	/
Immune response(%)	12w	/	/	/
	24w	100	92	/
Recurrence(%)	48w	0	/	5.4

In this study, we found that the combination of traditional Chinese medicine with CTX had a good clinical effect on patients with low baseline proteinuria and early pathological stage, while serum Anti-PLA2R antibody titers did not show a significant correlation with prognosis, which was considered to be related to the small sample size of this study. In patients who are immune remission without clinical remission, it is considered that proteinuria levels may decrease over the next 6 to 24 months. At the same time, for some patients with pre-existing renal impairment and/or mild decline in renal function at baseline, persistent proteinuria reflects the potential for residual structural destruction due to scarring rather than active disease.

Regarding side effects, CTX and traditional Chinese medicine work together to lower the risk of side effects including infection. During the 6-month course of treatment, there were only three infections, one of which was thought to be caused by an underlying illness. Furthermore, one patient experienced heart failure during treatment, which was thought to be related to the patient’s age and prior underlying conditions rather than the treatment regimen or NS. Two patients also experienced abnormal liver function during the follow-up period, which was resolved after stopping CTX. One patient developed nausea and diarrhea, which were not excluded from intolerance to taking traditional Chinese medicine. Because the lowest dose of CTX (50 mg once a day) was used in the same patient in this study, the cumulative dose during the treatment period was 9 g, and there was no high-dose GC therapy. In contrast, the current KDIGO guideline-recognized standard cytotoxicity-based regimen (the “Ponticelli regimen”) administers a cumulative dose of CTX of 15.75 g over 3 months and a cumulative dose of prednisone of 14.77 g over 3 months in 70 kg of individuals with normal renal function. This regimen is significantly lower than the dose range typically associated with gonadal toxicity, bladder toxicity, and malignancy (32).

A few other limitations of this research should be addressed. First, this is a single-arm study, so there is a lack of data from the control group, which could have provided more insight into the efficacy of this hormone-free regimen for medium-to high-risk PMN. Second, the detection rate of Anti-PLA2R antibodies was lower than expected, which may have limited the ability to fully evaluate the efficacy of this regimen.

This study suggests that, using the classic “Ponticelli regimen” from large RCTs as a reference, the efficacy and safety of CTX combined with the SLFX Formula are relatively favorable, making it a potential option for the clinical treatment of intermediate-to-high risk membranous nephropathy (MN) patients, particularly for elderly patients with contraindications to corticosteroid use or those with refractory disease. This study is an exploratory trial, and future large-scale randomized controlled trials will be conducted to further validate the efficacy of this regimen for intermediate-to-high-risk PMN. Additionally, animal experiments and cell experiments will be carried out to explore the relevant mechanisms of action of the traditional Chinese medicine SLFX Formula.

## 5 Conclusion

Our findings confirmed that the combination of traditional Chinese medicine SLFX Formula and low-dose CTX in the treatment of patients with intermediate-to-high risk PMN can achieve certain clinical efficacy in the short term, and reduce the adverse effects of CTX monotherapy.

## Data Availability

The raw data supporting the conclusions of this article will be made available by the authors, without undue reservation.
