# Supplementary material for: Low-dose cyclophosphamide combined with chinese herbal medicine Shuli Fenxiao formula for the treatment of intermediate-to-high risk primary membranous nephropathy
Source: Front Immunol. 2025 Apr 22;16:1543581. doi: 10.3389/fimmu.2025.1543581 (PMC12053483; doi:10.3389/fimmu.2025.1543581)
Supplement: Supplementary file 1 [file DataSheet1.pdf]

Supplementary Material

1. Figures

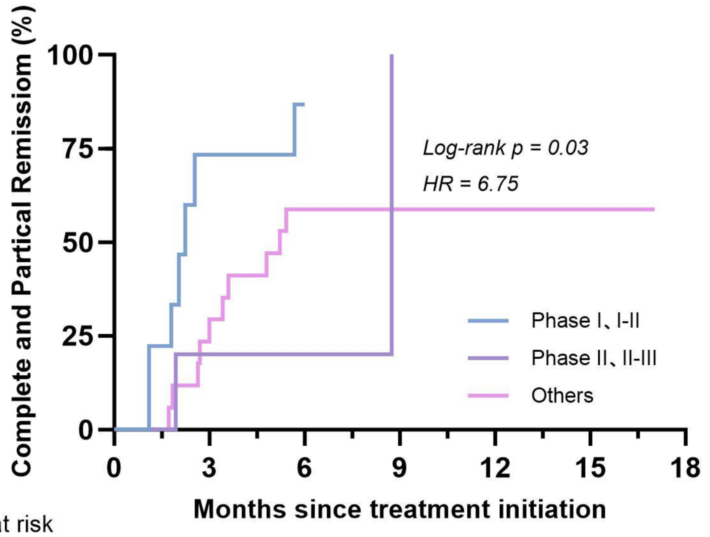

Number at risk							
Phase I、I-II	9	2	2	1	1	1	1
Phase II、II-III	5	4	3	0	0	0	0
Others	1	1	1	1	1	1	1

Figure S1. Kaplan-Meier curves for partial and complete remission by Pathologic stage.

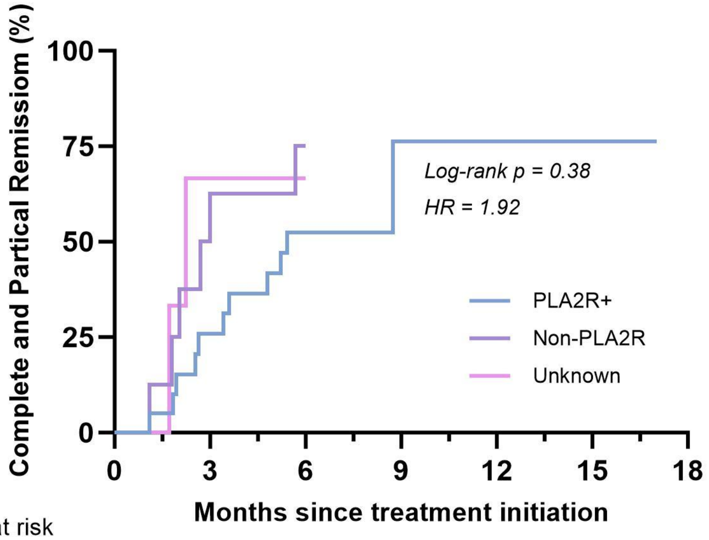

Number at risk							
PLA2R+	20	15	9	5	5	5	5
Non-PLA2R	8	3	2	0	0	0	0
Unknown	3	1	1	0	0	0	0

Figure S2. Kaplan-Meier curves for partial and complete remission by PLA2R correlation.

2. Specific form of the drug

Administration of Western medicine in clinical research:

Medicine	Specifications	Dosage	Manufacturer
Cyclophosphamide	50mg/ tablets	50mg qd	TongHuaMaoXiang Co., Ltd

Administration of Chinese medicine in clinical research:

Dongzhimen Hospital Granule Preparation:

Radix Astragali 50g(Huangqi);

Bitter Apricot 12g(Xingre);

Heartleaf Houttuynia 30g(Yuxingcao);

Magnolia Officinalis 15g(Houpo);

Rhizoma smilacis glabraea 30g(Tufuling);

Leech 9g(Shuizhi);

Bupleurum 30g(Chaihu);

Scutellaria 15g(Huangqin);

Chuanxiong 18g(Chuanxiong);

Cyathula Root 15g(Niuxi).

These herbs were decocted in water and administered orally as granules, with individualized modifications according to the patient's condition. For example, additional herbs were added for symptoms such as throat pain, cough with yellow sputum, sticky stools, severe lower limb edema, obvious blood stasis, and urinary heat and pain. The granules were taken once daily, dissolved in hot water, and taken 250 milliliters twice daily, in the morning and evening. This regimen was continued for 24weeks.
